# Genome Sequence of Historical *Bacillus anthracis* Strain Tyrol 4675 Isolated from a Bovine Anthrax Case in Austria

**DOI:** 10.1128/genomeA.00002-17

**Published:** 2017-03-09

**Authors:** Markus Antwerpen, Roman Wölfel, Gregor Grass

**Affiliations:** Bundeswehr Institute of Microbiology, Munich, Germany

## Abstract

In 1988, an outbreak of anthrax occurred among cattle in the Austrian state of Tyrol. Since then, Austria has been declared anthrax-free. Here, we report the draft genome sequence of one of these last outbreak strains, *Bacillus anthracis* Tyrol 4675, isolated from a diseased cow.

## GENOME ANNOUNCEMENT

The last outbreak of the zoonotic disease anthrax in Austria occurred in the state of Tyrol in 1988. *Bacillus anthracis* strain Tyrol 4675 was isolated from one of the diseased cows (1, 2). Strain Tyrol 4675 harbors both *B. anthracis* virulence plasmids pXO1 and pXO2, as confirmed by real-time PCR assays (1–4). Initial genotyping using canonical single-nucleotide polymorphisms (canSNP) (5) grouped strain Tyrol 4675 to branch B.Br. CNEVA (B2) (5). More specifically, it positions in subbranch B.Br.005/004 (6). This is the same subbranch as strain BF-1 (6, 7), isolated from the Bavarian northern foothills of the Alps neighboring Tyrol. Strains from this clade have been reported before from other central and western European countries, such as Germany or France, and this genotype appears to be autochthonous to this geographic region (5, 7, 8). Strain Tyrol 4675 represents the first representative genome sequence from the country of Austria.

Whole-genome shotgun (WGS) sequencing of *B. anthracis* Tyrol 4675 was performed by Illumina MiSeq sequencing technology (Illumina, Inc.) using Nextera version 3 with 2 × 300-bp chemistry. For the WGS library, 2 × 813,272 reads accumulating to a total of 464 Mb were generated. Burrows-Wheeler Aligner’s Smith-Waterman Alignment (BWA-SW) (9) was used for mapping to the *B. anthracis* Ames Ancestor chromosome and plasmids pXO1 and pXO2 (accession numbers NC_007530.2, NC_007322.2, and AE017335.3), respectively. The G+C content was calculated using an in-house python script.

The total length of the genome draft sequence of *B. anthracis* Tyrol 4675 was 5,227,565 bp, with 62.3-fold coverage for the chromosome, 145-fold for pXO1, and 102-fold for pXO2, respectively. The mean G+C content was 35.4%. For initial annotation, assembled contigs were submitted to the RAST Annotation Pipeline RASTk (10). The *B. anthracis* Tyrol 4675 draft genome contains 5,693 putative coding sequences (CDS). Within the genome annotation, there are 11 copies of genes for the 16S rRNA, the 5S rRNAs, and the 23S rRNA; 95 tRNA loci were identified.

### Accession number(s).

This whole-genome shotgun project has been deposited at DDBJ/EMBL/GenBank under the GenBank accession numbers CP018904 (pXO1), CP018905 (pXO2), and CP018903 (chromosome). The versions described in this paper are the first versions.
